# Orbitofrontal cortex connectivity is associated with food reward and body weight in humans

**DOI:** 10.1093/scan/nsab083

**Published:** 2021-07-14

**Authors:** Edmund T Rolls, Ruiqing Feng, Wei Cheng, Jianfeng Feng

**Affiliations:** Department of Computer Science, University of Warwick, Coventry CV4 7AL, UK; Institute of Science and Technology for Brain-Inspired Intelligence, Fudan University, Shanghai 200433, China; Oxford Centre for Computational Neuroscience, Oxford, UK; Department of Computer Science, University of Warwick, Coventry CV4 7AL, UK; Institute of Science and Technology for Brain-Inspired Intelligence, Fudan University, Shanghai 200433, China; Department of Computer Science, University of Warwick, Coventry CV4 7AL, UK; Institute of Science and Technology for Brain-Inspired Intelligence, Fudan University, Shanghai 200433, China; Key Laboratory of Computational Neuroscience and Brain-Inspired Intelligence, Fudan University, Ministry of Education, Shanghai 200433, China

**Keywords:** orbitofrontal cortex, food reward, resting-state functional connectivity, anterior cingulate cortex, obesity, body mass index

## Abstract

The aim was to investigate with very large-scale analyses whether there are underlying functional connectivity differences between humans that relate to food reward and whether these in turn are associated with being overweight. In 37 286 humans from the UK Biobank, resting-state functional connectivities of the orbitofrontal cortex (OFC), especially with the anterior cingulate cortex, were positively correlated with the liking for sweet foods (False Discovery Rate (FDR) *P* < 0.05). They were also positively correlated with the body mass index (BMI) (FDR *P* < 0.05). Moreover, in a sample of 502 492 people, the ‘liking for sweet foods’ was correlated with their BMI (*r* = 0.06, *P* < 10^−125^). In a cross-validation with 545 participants from the Human Connectome Project, a higher functional connectivity involving the OFC relative to other brain areas was associated with a high BMI (≥30) compared to a mid-BMI group (22–25; *P* = 6 × 10^−5^), and low OFC functional connectivity was associated with a low BMI (≤20.5; *P* < 0.024). It is proposed that a high BMI relates to increased efficacy of OFC food reward systems and a low BMI to decreased efficacy. This was found with no stimulation by food, so may be an underlying individual difference in brain connectivity that is related to food reward and BMI.

## Introduction

The primate including human orbitofrontal cortex (OFC) is involved in food reward, in that neurons in it respond to the sight, smell, taste and texture of food only when hunger is present and the food is rewarding, and functional magnetic resonance imaging (fMRI) activations in humans follow suit (Rolls *et al.*, 1989; Critchley and Rolls, 1996; Kringelbach *et al.*, 2003; Rolls, 2016b, 2019b; Rolls, 2020, 2021b). Further, activations of the OFC are linearly related to the subjective pleasantness of a food reward (de Araujo *et al.*, 2003; Kringelbach *et al.*, 2003; Grabenhorst and Rolls, 2008, 2009; Grabenhorst *et al.*, 2010). It has been proposed that a higher sensitivity to the food reward value of the sight, smell and taste of food represented in the OFC could contribute to obesity (Rolls, 2011, 2016b). At the other end of the spectrum, it is much less clear about whether a decreased sensitivity of the OFC food reward system contributes to some individuals having a low body weight. This is especially the case for anorexia, to which many other factors may contribute (Kaye, 2008; Kaye *et al.*, 2020). Much previous research on brain connectivity and obesity has focussed more on reduced functionality of brain systems related to executive function, rather than on an enhanced sensitivity of brain systems to food reward (Tregellas *et al.*, 2011; Kullmann *et al.*, 2012; Lips *et al.*, 2014; Garcia-Garcia *et al.*, 2015; Lowe *et al.*, 2019; Donofry *et al.*, 2020; Legget *et al.*, 2021). Social and cognitive factors can operate by top-down biassing of the reward-related representations in the OFC, with their origin in the dorsolateral prefrontal cortex (de Araujo *et al.*, 2005; Deco and Rolls, 2005; Grabenhorst and Rolls, 2008; Grabenhorst *et al.*, 2008; Rolls *et al.*, 2008; Grabenhorst and Rolls, 2010; Ge *et al.*, 2012; Luo *et al.*, 2013; Rolls, 2013, 2021a,b). For example, if a person is informed by another individual or by advertising that a food is in some way good or delicious, that can influence the eating behavior. One route by which this can happen is by top-down, cognitive and social, influences on the OFC food reward system, as described by (Rolls 2021b). In this context, a key aim of the present paper is to understand better the operation of food reward systems in the OFC and how they relate to body weight and body mass index (BMI).

To analyze whether there are differences in the functioning of the OFC that relate to whether individuals are overweight or underweight, in this investigation we measured the resting-state functional connectivity of the OFC and related this to food reward measured by the liking for sweet foods, and whether the participants were overweight or underweight, measured by their BMI. Functional connectivity is measured by the correlations between the blood oxygen level–dependent (BOLD) signal in different brain areas and reflects how strongly a pair of brain areas interacts. A high functional connectivity between brain areas provides an indication that there are strong influences between them, whether directly or indirectly. For example, a high functional connectivity of some brain areas may mean that they are more influenced by their inputs or that they influence their output regions more. Measurement of resting-state functional connectivity may provide a measure of whether the intrinsic brain circuitry is different in some individuals, even when it is not being used, for example, for food reward and the control of food intake.

Given this background, the first hypothesis that we wished to test was whether the resting-state functional connectivity of the OFC was higher in individuals who like sweet foods. The second hypothesis was whether those who like sweet foods have a higher BMI. The third hypothesis was whether a high functional connectivity of the OFC is associated with a higher BMI. There were also prior hypotheses about the other brain areas with which a high connectivity of the OFC would be of interest in relation to food reward and BMI. One is the anterior cingulate cortex, which has strong connections with the OFC, and is involved as a link from the reward-related OFC to output areas to perform actions to obtain rewards (Rolls, 2019a). This connectivity is highly correlated with sensation-seeking for reward (Wan *et al.*, 2020). Another brain region is the insula, which has a strong connectivity with the OFC, and parts of which may be involved in taste and in autonomic output to rewards (such as salivation to the sight of food) (Rolls, 2016a).

A feature of the present investigation is that functional connectivity and its relation to obesity were investigated in a very large number of individuals in the UK Biobank, with cross-validation with the Human Connectome Project (HCP), in order to promote robustness of the findings. Small resting-state fMRI studies with fewer than 50–100 participants may report false-positive results due to statistical fluctuations (Poldrack *et al.*, 2017; Gong *et al.*, 2019; Jiao *et al.*, 2020), and the present investigation helps to provide a quantitative assessment of not only whether effects are present but also of what their magnitude is to help guard against false-positive results in small studies. Another feature of the investigation is that brain function was being measured in the resting state without the sight or taste or food, to investigate whether there are differences in OFC functional connectivity even when food is not available. To our knowledge, this is the first time that discoveries of the type described here have been reported in large-scale studies, which are not subject to the vagaries of small-scale studies.

## Methods

### UK Biobank

#### Participants.

The dataset used for this investigation was selected from the September 2019 public data release from the UK Biobank, which includes a wide range of phenotypic information, as well as biological samples, for ∼500 000 participants. The UK Biobank sample used in these neuroimaging analyses included 37 286 participants [of whom 19 807 (53.12%%) were female; age range, 45–79 years]. The UK Biobank received ethical approval from the research ethics committee (REC reference 11/NW/0382). The present analyses were conducted under UK Biobank application number 1954. Written informed consent was obtained from each subject. The demographic characteristics of participants are summarized in Table 1.

**Table 1.** Demographic characteristics of the 37 286 UK Biobank participants

Characteristics	No. (%)
Age, mean (s.d.), years	61.74 (7.44)
Female	19 807 (53.12)
Townsend deprivation index, mean (s.d.), points	−1.90 (2.71)
Drinker status	
Prefer not to answer	9 (0.02)
Never	930 (2.49)
Previous	792 (2.12)
Current	35 544 (95.36)
Smoking status	
Never	22 782 (61.12)
Previous	12 179 (32.67)
Current	2,314 (6.21)
Education qualifications	
College or university degree	17 126 (46.56)
A levels/AS levels or equivalent	4,847 (13.18)
O levels/GCSEs or equivalent	7,101 (19.30)
CSEs or equivalent	1,505 (4.09)
NVQ or HND or HNC or equivalent	1,977 (5.37)
Other professional qualifications, e.g., nursing, teaching	1,823 (4.96)
None of the above	2,405 (6.54)
Liking for sweet foods, mean (s.d.), points	6.03 (2.10)
BMI, mean (s.d.), kg/m^2^	26.49 (4.37)

Analyses were performed for participants with both neuroimaging data and behavioral data concerning ‘liking for sweet foods’ (Field ID: 20732), which is liking for sweet foods measured on a 9-point scale from ‘extremely dislike’ to ‘extremely like’.

#### Imaging data collection and preprocessing.

The multi-modal imaging was collected using a standard Siemens Skyra 3T running VD13A SP4, with a standard Siemens 32-channel RF receive head coil. The resting-state functional brain imaging data used in this study included 22 331 participants and were obtained and processed by the UK Biobank. After quality controls and removing some participants without behavioral data, 4227 participants remained in the neuroimaging analysis. The details of the image acquisition are provided on the UK Biobank website in the form of a protocol (http://biobank.ctsu.ox.ac.uk/crystal/refer.cgi?id=2367). All the quality checking and data preprocessing procedures were conducted by the UK Biobank, and the details of the preprocessing are available on the UK Biobank website (http://biobank.ctsu.ox.ac.uk/crystal/refer.cgi?id=1977) and elsewhere (Miller *et al.*, 2016). Briefly, data preprocessing was carried out using FSL (FMRIB Software Library). All the data preprocessing procedures were performed by the UK Biobank team as described by Miller *et al.* (2016). The data preprocessing included correction for spatial and gradient distortions and head motion, intensity normalization and bias field removal, registration to the T1 weighted structural image, transformation to 2 mm Montreal Neurological Institute (MNI) space, and the FIX artifact removal procedure (Smith *et al.*, 2013; Navarro Schroder *et al.*, 2015). Finally, the head motion parameters were regressed out, and structured artifacts were removed by ICA + FIX processing (Independent Component Analysis followed by FMRIB’s ICA-based X-noiseifier; Griffanti *et al.*, 2014; Salimi-Khorshidi *et al.*, 2014). The data preprocessing pipeline developed by FMRIB (Oxford University Centre for Functional MRI of the Brain) used here has been widely used in resting-state fMRI studies (Navarro Schroder *et al.*, 2015; Smith *et al.*, 2015; Colclough *et al.*, 2017; Vidaurre *et al.*, 2018).

#### Statistical analyses.

Partial correlations were measured between the measure of ‘liking for sweet foods’ in the UK Biobank (Field ID: 20732) and the 94 × 94 Automated Anatomical Labeling 2 (AAL2) functional connectivity matrix across all the individuals, enabling statistically significant correlations to be identified in the 94 × 94 correlation matrix using FDR correction for multiple comparisons, with gender, age, education qualification, smoking status, drinker status, Townsend deprivation index, imaging center and head motion regressed out. The AAL2 atlas (Rolls *et al.*, 2015) was used because it defines a number of different OFC areas, with the full list of AAL2 areas shown in Supplementary Table S1, and because it has already proved useful in a number of investigations about the OFC (Du *et al.*, 2020; Hsu *et al.*, 2020; Rolls *et al.*, 2020; Wan *et al.*, 2020).

Similarly, partial correlations were measured between the BMI measure calculated from the data in the UK Biobank and the 94 × 94 AAL2 functional connectivity matrix across all the individuals, enabling statistically significant correlations to be identified in the 94 × 94 correlation matrix using FDR correction (Benjamini and Hochberg, 1995) for multiple comparisons, with gender, age, education qualification, smoking status, drinker status, Townsend deprivation index, imaging center and head motion regressed out.

The hypothesis for both these analyses is to find functional connectivity links in the brain that are positively correlated with the liking or BMI, as it is hypothesized that brain areas such as the OFC involved in food reward have activations and functional connectivities that are higher for high reward (Rolls, 2016b; Rolls *et al.*, 2020; Wan *et al.*, 2020).

### Human Connectome Project

#### Participants.

The dataset was selected from the March 2017 public data release from the HCP (*N* = 1200), WU-Minn Consortium. The sample included 1002 subjects (ages 22–37 years, 534 females) scanned on a 3T Siemens connectome-Skyra scanner. The WU-Minn HCP Consortium obtained full informed consent from all participants, and research procedures and ethical guidelines were followed in accordance with the Institutional Review Boards. The demographic characteristics of 545 participants in this study are summarized in Table 2.

**Table 2.** Demographic characteristics of the 545 HCP participants

Characteristics	No. (%)
Age, mean (SD), years	28.69 (3.75)
Female	310 (56.88)
Education, mean (SD), years	15 (1.77)
Drinker status	
Prefer not to answer	25 (4.59)
Never	80 (14.68)
Previous	255 (46.79)
Current	185 (33.94)
Smoking status	
Never	295 (54.13)
Previous	160 (29.36)
Current	90 (16.51)
BMI	
Low BMI (BMI ≤ 20.5)	83 (15.23)
Mid BMI (22 < BMI < 25)	256 (44.99)
High BMI (BMI ≥ 30)	206 (36.20)

The HCP participants were divided into three groups, using the principle that there should be >80 participants in each of a low, mid and high BMI group, with a gap of BMI between each group to facilitate comparison between the different groups. The BMI is defined as the weight (in kg)/height^2^ (in meters). Using these criteria, the high BMI group with BMI  ≥30 contained 206 participants (111 females), the mid group with BMI between 22 and 25 contained 256 participants (137 females) and the low BMI group with BMI ≤20.5 contained 83 participants (62 females). These particular values for the BMI for the different groups were selected to have sufficient participants in each group for the analyses, to allow a gap between the BMIs of the groups to facilitate statistical comparison and to provide data from groups with low, mid and high BMIs from the population in the dataset. The collection and preprocessing of the data are provided on the HCP website (http://www.humanconnectome.org/), together with information about ethical permission and informed consent.

#### Data collection and preprocessing.

The data collection and preprocessing were performed by the HCP. The participants included in this sample were scanned on a 3T connectome-Skyra scanner (Siemens). Data preprocessing was carried out using FSL (FMRIB Software Library), FreeSurfer and the Connectome Workbench software. All the data preprocessing procedures were performed by the HCP as described in detail elsewhere (Glasser *et al.*, 2013). The data preprocessing included correction for spatial and gradient distortions and head motion, intensity normalization and bias field removal, registration to the T1 weighted structural image, transformation to 2 mm MNI space and the FIX artifact removal procedure (Smith *et al.*, 2013; Navarro Schroder *et al.*, 2015). Finally, the head motion parameters were regressed out and structured artifacts were removed by ICA + FIX processing (Independent Component Analysis followed by FMRIB’s ICA-based X-noiseifier; Griffanti *et al.*, 2014; Salimi-Khorshidi *et al.*, 2014). The data preprocessing pipeline developed by FMRIB (Oxford University Centre for Functional MRI of the Brain) used here has been widely used in resting-state fMRI studies (Navarro Schroder *et al.*, 2015; Smith *et al.*, 2015; Colclough *et al.*, 2017; Vidaurre *et al.*, 2018). The first 20 volumes were discarded to suppress equilibration effects, and participants without the full 1200 time points in four resting-state runs were also removed from the following analysis. The resulting time courses were used for the construction and analysis of the functional connectivity brain network.

#### Construction of the whole-brain functional connectivity network.

After the preprocessing, the gray matter of the whole brain was divided into 94 regions using the AAL2 atlas (Rolls *et al*., 2015). This atlas was chosen because it has a useful parcellation of the OFC based on (Chiavaras and Petrides, 2000) and because there is evidence on the connectivity of each of the OFC divisions with each other and with other brain regions, using both direct connectivity (Hsu *et al*., 2020) and functional connectivity (Du *et al*., 2020). Based on the AAL2 atlas, the time series were extracted by determining the mean of the signals of all voxels within each region across 490 time points. The whole-brain functional network (94 × 94 regions with 4371 links) was established by calculating the Pearson’s correlation between the BOLD signal for all pairs of brain regions for each individual participant, followed by *z* transformation to improve normality (Finn *et al*., 2015; Rosenberg *et al*., 2016). The anatomical regions in the AAL2 atlas are shown in Supplementary Table S1. Age, gender, education years, drinking status, smoking status and head motion were regressed out in the analyses.

#### Statistical analyses.

**Fig. 3.**
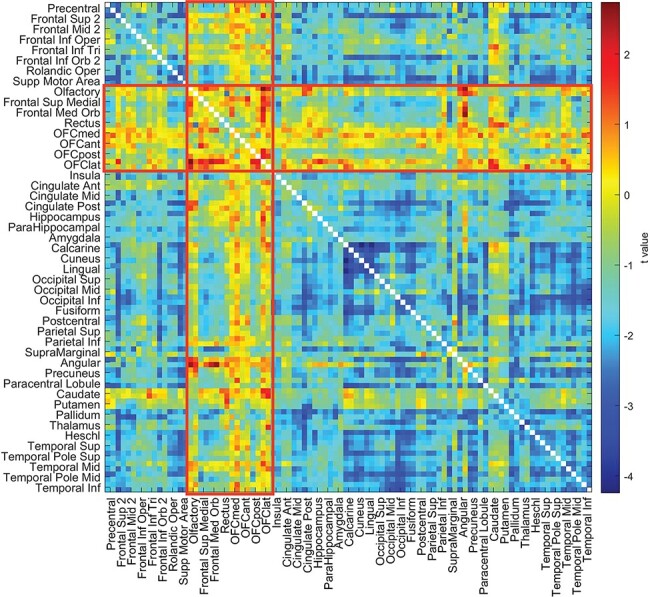
The resting-state functional connectivities involving the OFC are higher relative to other functional connectivities in the high BMI group than the mid BMI group in the HCPt dataset. For the HCP dataset, the *t* values for the resting-state functional connectivity differences for the high BMI minus the mid BMI participants are shown, with age, gender, education years, drinking status, smoking status and head motion regressed out. The areas referred to are olfactory (an area just posterior to the OFC in the AAL2 atlas) to the lateral OFC (OFClat) in the AAL2 atlas, and their connectivities with all other brain areas are within the red rectangles. The BMI groups and the numbers of participants in each are shown in Figure 5. The statistics are provided in the text and compared connectivities within the red rectangles for olfactory to OFClat with those outside the red rectangles.

**Fig. 5.**
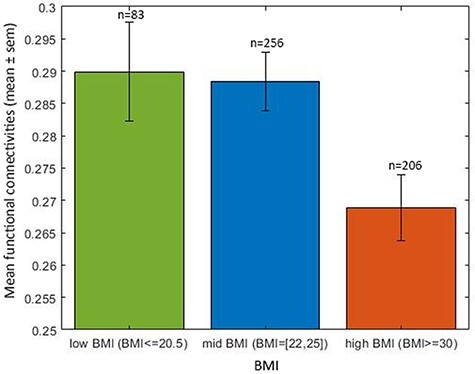
High BMI is associated with low mean functional connectivity. The relation between the mean FC across all cortical areas for the three groups with different BMI for the HCP. The high BMI group has a lower mean functional connectivity compared to the mid BMI group (*t* = −2.9, Cohen’s *d* = −0.13, df = 460, *P* = 4.3 × 10^−3^), with age, gender, education years, drinking status, smoking status and head motion regressed out. The numbers of participants in each group are shown.

t tests were performed to test for differences of the 94 × 94 functional connectivity matrix between the different groups, with age, gender, education years, drinking status, smoking status and head motion regressed out. This resulted in a single 94 × 94 t matrix for the *t* values of the differences between each of the functional connectivities for the difference between, for example, the high BMI group compared to the mid BMI group. To test whether the functional connectivities were different for the high BMI group and the mid BMI group, the difference of the mean OFC functional connectivity from the mean Other functional connectivity was calculated for each participant and then the difference of these was compared using a t-test across participants between the two BMI groups. This controlled for any possible difference of the mean functional connectivity between the three BMI groups, which was tested (and found to be present as shown in Figure 5). The OFC functional connectivities were those within the OFC region of interest (OFC ROI1, from Olfactory to OFClat in the AAL2 atlas) together with the connectivities of these OFC regions with other brain areas. The Other functional connectivities were those not involving areas in the OFC ROI1. A similar comparison was performed to test whether the OFC functional connectivities were different from all the Other functional connectivities when comparing the low BMI group to the mid BMI group. The OFC ROI2 was defined as AAL2 areas OFCant to OFClat (see Supplementary Table S1). These ROIs for the OFC areas were selected based on very extensive evidence from primate single neuron recording and human fMRI that these brain regions are activated by stimuli such as the sight, smell, taste and texture of food when they are rewarding and are subjectively pleasant (Rolls *et al.*, 1989; Critchley and Rolls, 1996; Kringelbach *et al.*, 2003; Rolls, 2014; 2015; Rolls, 2016b, 2019b; 2020; Rolls, 2021a,b), with ROI1 extended to include areas known to be connected with the OFC (Du *et al.*, 2020; Hsu *et al.*, 2020) that also had a high functional connectivity in the high BMI *vs* mid BMI groups in Figure 3.

## Results

### UK Biobank

#### Correlation between resting-state functional connectivity and the liking for sweet food.

**Fig. 1.**
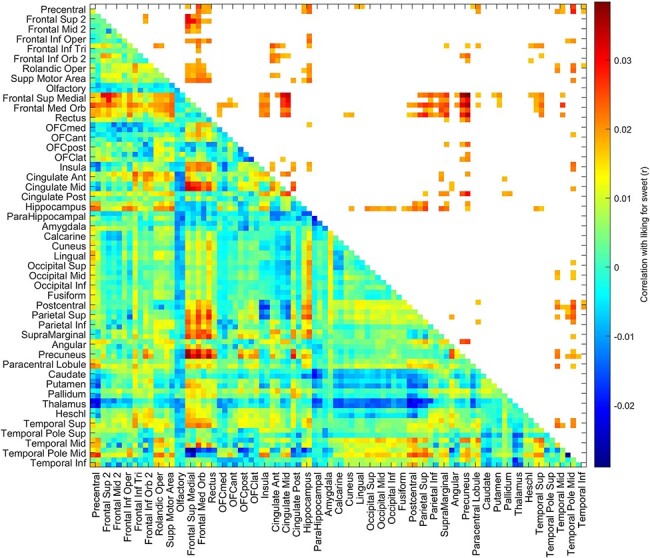
The liking for sweet foods is correlated with functional connectivities of the VMPFC (in this case gyrus rectus, FrontalMedOrb and FrontalSupMed from the AAL2 atlas). For the UK Biobank, the correlation between the ‘liking for sweet foods’ and the resting-state functional connectivities between all AAL2 brain areas. The lower left triangle shows the Pearson’s correlations with the 94 × 94 functional connectivities, and the upper right triangle shows those that were significantly higher after FDR correction (*P* < 0.05 with a two-tailed test). A list of the AAL2 areas and their abbreviations is provided in Supplementary Table S1. Frontal_Med_Orb with Rectus corresponds to the VMPFC and Frontal_Sup_Medial includes cortex just superior to this. The OFC areas are OFCmed to OFClat. There were 37 286 participants.

The correlations between the ‘liking for sweet foods’ and the resting-state functional connectivities between all AAL2 brain areas are shown in Figure 1, with the top 50 correlated links in Table 3. From Figure 1, it is evident that there is a set of ventromedial prefrontal cortex (VMPFC) areas (Frontal_Med_Orb with Rectus that corresponds to the VMPFC and Frontal_Sup_Medial just superior to this) whose connectivity with the anterior and midcingulate cortex, and the insula, is correlated with the reported liking for sweet foods. The functional connectivities of the same VMPFC medial prefrontal areas with many other brain areas also had high correlations with the reported ‘liking for sweet foods’. This is confirmed by the links shown in Table 4, many of which involved VMPFC and related areas (Frontal_Med_Orb; together with Frontal_Sup_Medial just superior to this) with the anterior cingulate cortex. Other links that feature in the table involve the precuneus, parietal areas, including the angular, supramarginal and inferior parietal cortex, the middle frontal gyrus, and the temporal lobe.

**Table 3.** The 50 most significant functional connectivity links positively correlated with the liking for sweet foods in the UKB (*N* = 37 286)

Region_1	Region_2	*r* Value	*P* value	Region_1	Region_2	*r* Value	*P* value
Frontal_Sup_Medial_L	Precuneus_R	0.04	6.10E-12	Frontal_Med_Orb_L	SupraMarginal_R	0.03	2.41E-06
Frontal_Sup_Medial_L	Precuneus_L	0.04	1.02E-10	Cingulate_Mid_R	Cingulate_Post_L	0.03	2.56E-06
Frontal_Med_Orb_L	Precuneus_R	0.04	2.43E-10	Frontal_Mid_2_L	Frontal_Sup_Medial_L	0.03	2.65E-06
Rectus_L	Precuneus_R	0.03	7.01E-10	Postcentral_R	Temporal_Pole_Mid_R	0.03	2.67E-06
Frontal_Sup_Medial_L	Cingulate_Mid_R	0.03	1.19E-09	Angular_L	Precuneus_R	0.03	2.95E-06
Frontal_Sup_Medial_L	Cingulate_Mid_L	0.03	1.01E-08	Hippocampus_R	Parietal_Sup_R	0.03	2.96E-06
Frontal_Med_Orb_L	Precuneus_L	0.03	1.20E-08	Frontal_Sup_Medial_R	Precuneus_L	0.03	2.96E-06
Frontal_Sup_2_L	Frontal_Sup_Medial_L	0.03	1.35E-08	Frontal_Med_Orb_R	Precuneus_R	0.03	3.42E-06
Frontal_Sup_Medial_R	Cingulate_Mid_R	0.03	1.80E-08	Precentral_L	Frontal_Med_Orb_L	0.03	3.53E-06
Frontal_Sup_2_R	Frontal_Sup_Medial_L	0.03	2.84E-08	Frontal_Med_Orb_L	Parietal_Inf_L	0.03	3.66E-06
Frontal_Sup_Medial_R	Precuneus_R	0.03	4.48E-08	Frontal_Med_Orb_R	SupraMarginal_L	0.03	3.76E-06
Frontal_Sup_2_R	Frontal_Sup_Medial_R	0.03	4.82E-08	Precentral_L	Temporal_Pole_Mid_R	0.03	4.72E-06
Cingulate_Post_L	Precuneus_R	0.03	7.75E-08	Frontal_Med_Orb_L	Parietal_Sup_L	0.02	6.66E-06
Frontal_Med_Orb_L	Cingulate_Mid_R	0.03	2.12E-07	Frontal_Sup_Medial_L	Parietal_Inf_L	0.02	6.84E-06
Rectus_L	Parietal_Sup_R	0.03	2.28E-07	Frontal_Inf_Tri_L	Cingulate_Ant_L	0.02	7.56E-06
Precentral_R	Temporal_Pole_Mid_R	0.03	3.67E-07	Cingulate_Post_L	Precuneus_L	0.02	8.29E-06
Rectus_L	SupraMarginal_R	0.03	5.22E-07	Angular_L	Precuneus_L	0.02	8.31E-06
Rectus_L	Precuneus_L	0.03	5.25E-07	Frontal_Sup_2_R	Frontal_Med_Orb_L	0.02	8.34E-06
Frontal_Med_Orb_L	Parietal_Sup_R	0.03	1.23E-06	Rectus_L	Cingulate_Mid_L	0.02	1.03E-05
Frontal_Sup_Medial_L	SupraMarginal_R	0.03	1.73E-06	Hippocampus_R	SupraMarginal_L	0.02	1.05E-05
Rectus_L	Cingulate_Mid_R	0.03	1.85E-06	Parietal_Sup_R	Temporal_Pole_Mid_R	0.02	1.11E-05
Rectus_L	Parietal_Sup_L	0.03	1.96E-06	Frontal_Med_Orb_L	SupraMarginal_L	0.02	1.15E-05
Frontal_Med_Orb_L	Temporal_Mid_R	0.03	2.05E-06	OFCpost_L	Precuneus_R	0.02	1.28E-05
Frontal_Med_Orb_L	Cingulate_Mid_L	0.03	2.07E-06	Supp_Motor_Area_R	Frontal_Med_Orb_L	0.02	1.54E-05
Frontal_Sup_Medial_R	Cingulate_Mid_L	0.03	2.16E-06	Supp_Motor_Area_R	Hippocampus_R	0.02	1.64E-05

The *P* values shown are uncorrected for multiple comparisons, and the value corresponding to FDR *P* < 0.05 is *P* = 5.0 × 10^–3^.

**Table 4.** The 50 most significant functional connectivity links positively correlated with BMI in the UKB (*N* = 37 286)

Region_1	Region_2	*r* Value	*P* value	Region_1	Region_2	*r* Value	*P* value
Supp_Motor_Area_R	Parietal_Inf_R	0.06	3.60E-31	Precentral_R	Temporal_Sup_R	0.04	2.55E-14
Frontal_Med_Orb_R	Caudate_L	0.05	1.07E-21	Frontal_Med_Orb_R	Angular_L	0.04	2.74E-14
Frontal_Inf_Tri_L	OFCant_L	0.05	3.41E-21	Frontal_Med_Orb_L	Angular_L	0.04	8.74E-14
Supp_Motor_Area_L	Parietal_Inf_R	0.05	4.11E-20	Olfactory_L	Frontal_Sup_Medial_L	0.04	8.75E-14
Frontal_Inf_Orb_2_R	OFCant_R	0.05	1.12E-19	Postcentral_R	Parietal_Inf_R	0.04	1.14E-13
Precentral_L	Parietal_Inf_R	0.05	3.24E-19	Cingulate_Ant_L	Angular_L	0.04	1.75E-13
Frontal_Inf_Orb_2_L	OFCant_L	0.05	4.14E-18	Olfactory_L	Angular_R	0.04	2.22E-13
Precentral_R	Parietal_Inf_R	0.05	7.44E-18	Frontal_Sup_2_L	Cingulate_Ant_L	0.04	3.16E-13
Frontal_Med_Orb_L	Caudate_L	0.04	4.40E-17	Frontal_Sup_Medial_R	Caudate_L	0.04	3.32E-13
Frontal_Sup_2_L	Frontal_Med_Orb_R	0.04	5.93E-17	Frontal_Inf_Oper_L	SupraMarginal_R	0.04	3.35E-13
Frontal_Sup_Medial_R	Cingulate_Ant_L	0.04	1.91E-16	Frontal_Sup_Medial_L	Cingulate_Ant_L	0.04	5.37E-13
Olfactory_L	Angular_L	0.04	2.76E-16	Precentral_R	Parietal_Inf_L	0.04	5.39E-13
Frontal_Med_Orb_R	Cingulate_Ant_R	0.04	5.31E-16	Parietal_Inf_R	Paracentral_Lobule_L	0.04	8.03E-13
Frontal_Inf_Orb_2_L	OFCant_R	0.04	5.56E-16	Frontal_Mid_2_L	Frontal_Med_Orb_R	0.04	1.21E-12
Postcentral_L	Parietal_Inf_R	0.04	1.52E-15	Frontal_Inf_Tri_R	Supp_Motor_Area_R	0.04	2.14E-12
Supp_Motor_Area_L	Cingulate_Ant_L	0.04	3.93E-15	Parietal_Sup_L	Parietal_Inf_R	0.04	2.39E-12
Olfactory_L	Frontal_Sup_Medial_R	0.04	4.99E-15	Rectus_L	OFCant_L	0.04	3.92E-12
Frontal_Med_Orb_R	Caudate_R	0.04	5.00E-15	Postcentral_R	Parietal_Inf_L	0.04	4.73E-12
Frontal_Sup_Medial_R	Cingulate_Ant_R	0.04	6.16E-15	Frontal_Mid_2_L	Frontal_Med_Orb_L	0.04	4.99E-12
Frontal_Med_Orb_R	Cingulate_Ant_L	0.04	8.58E-15	Frontal_Sup_2_R	Supp_Motor_Area_L	0.04	5.17E-12
Frontal_Med_Orb_L	Cingulate_Ant_L	0.04	9.81E-15	Frontal_Mid_2_L	Angular_L	0.04	6.38E-12
Frontal_Sup_2_L	Angular_L	0.04	1.40E-14	Frontal_Inf_Tri_L	OFCant_R	0.04	8.19E-12
Frontal_Inf_Oper_R	SupraMarginal_L	0.04	1.82E-14	Frontal_Sup_2_R	Frontal_Med_Orb_R	0.04	1.10E-11
SupraMarginal_L	SupraMarginal_R	0.04	2.00E-14	OFCant_L	Angular_L	0.04	1.36E-11
Frontal_Sup_Medial_R	OFCant_R	0.04	2.44E-14	Supp_Motor_Area_R	Parietal_Inf_L	0.04	1.43E-11

The *P* values shown are uncorrected for multiple comparisons, and the value corresponding to FDR *P* < 0.05 is *P* = 6.1 × 10^−3^.

#### Correlation between the liking for sweet food and the BMI.

In a sample of 502 492 people in the UK Biobank, the ‘liking for sweet foods’ was significantly correlated with their BMI (*r* = 0.06, *P* < 10^−125^). (The larger number for this analysis is because there are data on BMI and the liking for sweet foods in more UK Biobank participants than had neuroimaging data. For the subset of 37 286 participants with neuroimaging data and with measurement for ‘liking for sweet food’, the correlation between ‘liking for sweet food and BMI was 0.06, *P* < 10^−32^.)

#### Correlation between resting-state functional connectivity and the BMI.

**Fig. 2.**
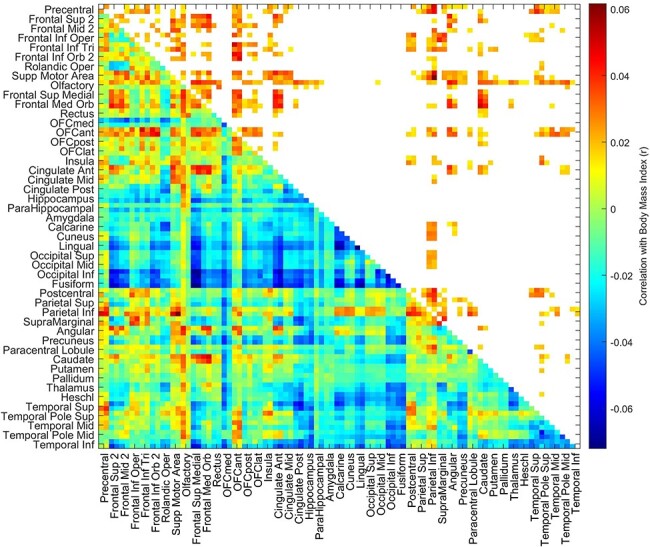
BMI is correlated with functional connectivities of the VMPFC and anterior OFC (OFCant from the AAL2 atlas). For the UK Biobandatak, the correlation between the BMI and the resting-state functional connectivities between all AAL2 brain areas. The lower left triangle shows the Pearson’s correlations with the 94 × 94 functional connectivities, and the upper right triangle shows those that were significantly higher after FDR correction (*P* < 0.05 with a two-tailed test). There were 37 286 participants.

The correlations between the BMI and the resting-state functional connectivities between all AAL2 brain areas are shown in Figure 2, with the top 50 functional connectivity links positively correlated with BMI shown in Table 4. From Figure 2, it is evident that the key part of the VMPFC (Frontal_Med_Orb; together with Frontal_Sup_Medial just superior to this) has functional connectivity with the anterior cingulate cortex that is significantly correlated with the BMI. (This was confirmed in that the functional connectivities between areas FrontalSupMed to OFClat had higher correlations with BMI than did the connectivities not involving these brain regions, *t* = 50.6, Cohen’s *d* = 0.53, *P* < 10^−325^, df = 7756.) More details are provided by the links shown in Table 4, many of which involved VMPFC and related areas (Frontal_Med_Orb; together with Frontal_Sup_Medial just superior to this) with the anterior cingulate cortex. Other links that feature in the table involve the precuneus, parietal areas, including the angular, supramarginal and inferior parietal cortex, and the middle frontal gyrus. The positive links were selected for Table 4 and Figure 2, because the hypothesis is that high functional connectivities of the ventromedial prefrontal/OFC and related areas are related to a high BMI and because a high BMI is associated with a tendency for functional connectivities to be generally lower, as shown in Figures 2 and 5.

### Human Connectome Project

#### OFC functional connectivity is higher relative to other brain areas in the high BMI group compared to the mid BMI group.

The resting-state functional connectivity differences between the high BMI and mid-weight participants, with age, gender, education years, drinking status, smoking status and head motion regressed out, are shown in Figure 3. It appears that functional connectivity differences between the high and mid BMI groups have higher *t* values between the OFC ROI1 areas than between the other AAL2 areas. We tested this, whether *ex hypothesi* the functional connectivity between the OFC areas relative to the other areas was higher in the high BMI group than the mid BMI group, as shown by the *t* values of the differences. This was performed by taking for each participant the mean value of the OFC functional connectivities with themselves and all other brain areas, and the mean value of the functional connectivities of all the other brain areas with each other, and using a t-test to compare the differences of these two values across all participants in the high BMI group *vs* the mid BMI group. This showed that the OFC functional connectivities relative to the other functional connectivities were higher in the high than the mid BMI groups (*t* = 4.05, Cohen’s *d* = 0.10, df = 462, *P* = 6 × 10^−5^). This design involving a within-subject difference of the OFC from the other functional connectivities was useful, as it factors out any overall decrease of functional connectivity with a high BMI evident in Figure 5. For this analysis, the OFC ROI1 definition included all areas from Olf to OFClat in the AAL2 atlas (see Figure 3, in which these brain regions are outlined with red rectangles). This analysis thus confirms that the OFC functional connectivities are higher relative to the other functional connectivities in the high BMI group than in the mid BMI group.

#### OFC functional connectivity is lower relative to other brain areas in the low BMI group compared to the mid BMI group.

**Fig. 4.**
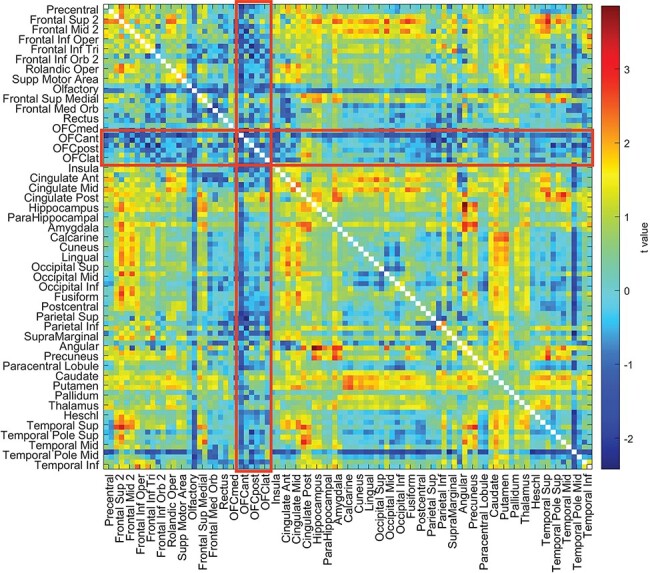
The resting-state functional connectivities involving the OFC are lower relative to other functional connectivities in the low BMI group than the mid BMI group in the HCP dataset. For the HCP dataset, the *t* values for the resting-state functional connectivity differences for the low BMI minus the mid BMI participants are shown, with age, gender, education, drinking frequency, smoking status and head motion regressed out. The areas referred to are anterior OFC (OFCant) to lateral OFC (OFClat) in the AAL2 atlas, and their connectivities with all other brain areas are within the red rectangles. The BMI groups and the numbers of participants in each are shown in Figure 5. The statistics are provided in the text and compared connectivities within the red rectangles for OFCant to OFClat with those outside the red rectangles.

The resting-state functional connectivity differences between the low BMI and mid-weight participants are shown in Figure 4. This shows that at least some OFC regions had lower functional connectivities relative to other brain areas in the low BMI group than in the mid BMI group, with the main OFC areas involved from OFCant to OFClat. Using a statistical analysis similar to that used for Figure 3, it was found that these OFC functional connectivities are lower relative to the other functional connectivities in the low BMI group than in the mid BMI group (*t* = −2.27, Cohen’s *d* = −0.10, df = 340, *P* < 0.024). For this analysis, the OFC definition included all areas from OFCant to OFClat in the AAL2 atlas (see Figure 4, in which these brain regions are outlined with red rectangles). For this analysis, the low BMI group included 83 individuals with a BMI ≤20.5, to increase a little the separation of the BMI between the low and mid BMI groups (with 259 individuals).

It was noticeable that the functional connectivities of brain areas other than those related to the OFC were negatively correlated with the BMI in the HCP dataset, and indeed, it was found that the mean functional connectivity across the whole brain was lower in the high BMI group compared to the mid BMI group, as shown in Figure 5. It was because of what is shown in Figure 5 that it was useful in the HCP analyses to compare the functional connectivities of the OFC areas with the functional connectivities of other brain areas.

## Discussion

The first key finding was that the resting-state functional connectivity between the VMPFC /OFC (VMPFC–OFC) and especially the anterior cingulate cortex was positively correlated with the liking for sweet foods (Figure 1). What is especially interesting about this is that the functional connectivity was measured in the resting state, when food was not present as a reward stimulus. There is much evidence that the OFC has neurons that respond to the reward value of the sight, smell taste and oral texture of food and that activations of the medial OFC in humans are related to the reward value of all of these sensory aspects of food (Rolls, 2015, 2016b, 2019b, 2021b; Rolls *et al.*, 2020), but the evidence that the functional connectivity of these areas is higher in those who report a high liking for sweet foods provides an indication that these brain food reward systems may be more strongly connected in individuals who like sweet foods. The stronger connections in this food reward system in some individuals may, we suggest, be a factor that drives some individuals more toward eating foods, which may not be limited to sweet foods but could extend to other types of food. The connectivity of the VMPFC/medial OFC with the anterior cingulate cortex is of especial interest, for the cingulate cortex is involved in actions, and in particular in associating actions (using information received from the parietal cortex) with rewards (received from the OFC/VMPFC) (Rolls, 2019a). Consistent with this, there are strong connections between the OFC and anterior cingulate cortex (Du *et al.*, 2020; Hsu *et al.*, 2020), and it is possible to predict from the strength of these connections human sensation-seeking behavior, which involves being highly motivated to seek out rewards (Wan *et al.*, 2020). Other areas with a high functional connectivity with the VMPFC in those who liked sweet included the precuneus (which is implicated in the sense of self; Cavanna and Trimble, 2006; Freton *et al.*, 2014); parts of the parietal cortex implicated in language including the angular, supramarginal and inferior parietal areas (Rolls, 2021a); the middle frontal gyrus (involved in executive function) and the temporal lobes (involved in visual and semantic representations) (Rolls, 2021a). It was further of interest that the VMPFC–OFC area related to the liking for food extended into other cortex in the medial prefrontal cortex (FrontalSupMed in the AAL2 atlas), which is an area with strong connections with the VMPFC (Du *et al.*, 2020; Hsu *et al.*, 2020).

The second key finding was that this same extended VMPFC–OFC area has functional connectivity positively correlated with BMI (Figure 2). This is consistent with the hypothesis that a high sensitivity to food reward (in this case measured by the liking for sweet foods) in this extended VMPFC system is associated with a higher BMI. Indeed, we suggest that this provides evidence that high liking for food in this food reward circuitry over-drives food intake to result in being overweight. Again, it is of great interest that the association between the connectivity of this extended VMPFC–OFC system and BMI can be revealed even in the resting state when no food is present, providing an indication that increased connectivity in this brain system is related to increased liking for foods and increased BMI. Of course these findings are associations and do not prove causality, but it is a long-established hypothesis that too much sensitivity to the sensory stimuli that produce food reward is a factor in obesity (Rolls, 2014, 2016b).

These discoveries in the UK Biobank dataset were cross-validated in the HCP dataset but also extended as described next, in that in addition to measuring correlations between VMPFC/OFC functional connectivity (relative to other brain areas) and body weight (which were significant), we were able to go beyond correlations and examine separately the situations in groups with a high BMI from those with a low BMI, with the latter of interest in relation to whether underconnectivity of the same system is related to a low BMI produced potentially by less food reward and therefore eating. This latter hypothesis was supported.

The finding shown in Figure 3 from the HCP that the functional connectivities of the OFC are higher in the high BMI group relative to the other FCs is consistent with the hypothesis that increased efficacy in the OFC food reward system may be a driving factor that contributes to increased eating and a higher BMI.

The finding shown in Figure 4 from the HCP that the FCs of the OFC (and especially the part involving OFCant, OFCpost and OFClat) are lower in the low BMI group relative to the other FCs is consistent with the hypothesis that decreased efficacy in the OFC food reward system may be a driving factor that contributes to decreased eating and a lower BMI. Although this low BMI group was not selected to have a diagnosis of anorexia, the findings here do provide evidence from the HCP dataset that in at least low BMI individuals (BMI < 20.5), the OFC, involved in food reward, does have decreased efficacy. That could relate to the low BMI if these individuals find food less rewarding and eat less. Indeed, for the low BMI group, our hypothesis is that the low functional connectivities of the OFC regions are related to the low BMI because of lower food reward, with reasoning similar to that provided above.

The results in Figures 3 and 5 suggest that a high BMI may be associated on average with somewhat lower functional connectivities averaged across all brain regions. This was tested by comparing the mean FC across all brain areas with the BMI, and the mean functional connectivity was lower in the high BMI group compared to the mid BMI group, with a significant difference in the HCP (*t* = −2.9, Cohen’s *d* = −0.13, df = 460, *P* = 4.3 × 10^−3^) and in the UK Biobank (UKB) (*t* = −13.4, Cohen’s *d* = −0.10, df = 16 673, *P* = 1.1 × 10^−40^). Because there are some differences of mean functional connectivity in the groups with different BMIs in this investigation (Figure 5), it was an important part of the statistical design of this investigation that the functional connectivities of the OFC ROI were compared with all the other functional connectivities within the same individuals, as a direct comparison of the functional connectivities of the OFC ROIs between groups with different BMIs could be influenced by the difference in mean functional connectivity between the groups with different BMIs that are shown in Figure 5.

An interesting finding from the HCP investigation was that the functional connectivities of the OFC ROI with the anterior cingulate cortex were high relative to the FCs of the OFC with other brain regions. This is consistent with the hypothesis that food reward signals from the OFC provide an extra strong input to the anterior cingulate cortex, which provides a route to implementing actions to obtain the food reward (Rolls, 2019a, 2021a,b). Consistent with this, sensation-seeking for rewards is associated with a higher functional connectivity between the reward-related medial OFC and the anterior cingulate cortex (Wan *et al.*, 2020). It has also been reported that those who binge eat compared to a control group show greater activation to food stimuli of the anterior cingulate cortex (Geliebter *et al.*, 2016). The pregenual anterior cingulate cortex, which receives inputs from the medial OFC (Du *et al.*, 2020; Hsu *et al.*, 2020), shares with the medial OFC activation produced by many rewarding stimuli, including olfactory and food-related stimuli, and correlations with the subjective pleasantness of these stimuli (Kringelbach *et al.*, 2003; Grabenhorst and Rolls, 2011; Rolls, 2014, 2019a,b).

Another interesting finding was that in the HCP the OFC ROI had a higher functional connectivity with the olfactory tubercle region in the high BMI group, which is part of the ventral striatum (Figures 3 and 5). The ventral striatum is a region activated by rewards and provides another route for the OFC to influence behavior (Rolls, 2017, 2019b, 2021a,b), as does the connectivity with the caudate nucleus and putamen also evident in Figures 3 and 4.

A further interesting finding in the HCP was that the OFC ROI had a higher functional connectivity with a number of other frontal cortex areas in the high BMI group, including the superior and middle frontal gyri and the superior medial prefrontal area (FrontalSupMed) (Figures 3 and 5). These are brain areas implicated in executive control and planning (Rosati, 2017; Shallice and Cipolotti, 2018; Fiske and Holmboe, 2019; Rolls, 2021a), and a hypothesis is that these prefrontal executive function areas receive a stronger food reward input from the OFC in high BMI individuals, which may influence the outcome of their plans.

Given the association described here between resting-state functional connectivity of the OFC and related regions and the liking for food and potentially thereby BMI, the differences between individuals might reflect genetic differences with some individuals more influenced by food reward, with individual variability in the value of different specific types of reward a driving factor in evolution by natural selection (Rolls, 2014). In a complementary way, top-down social or cognitive influences might bias the OFC reward systems and contribute to individual differences (Rolls, 2021b). For the findings from the UK Biobank, one of the covariates of no interest regressed out of the analysis was the Townsend deprivation index. The Townsend deprivation index reflects a range of measures of socioeconomic status, and so the results described here do not reflect what is measured by the Townsend deprivation index. Further research will of course be of interest to assess different contributions to the associations described here between functional connectivity, food reward and BMI.

In considering possible limitations, it is noted that with large datasets, the results can become highly significant. We therefore provide either *r* values or where t tests have been performed Cohen’s *d* values, in order to show the effect sizes. The effect sizes were in the range *d* = 0.1–0.53 (Figure 2). However, in fact, the large datasets used in this research are a great strength of this study, for they provide a firm foundation for research in this area that is not subject to the problems and possible false positives of small resting-state functional connectivity studies. We further note that the results described are quite selective, in that as shown in the top right parts of Figures 1 and 2, only a small proportion of the functional connectivities were significantly correlated after FDR correction for multiple comparisons with the liking for sweet food or with BMI.

Finally, we emphasize that this investigation is a very large study, with 37 286 participants with functional neuroimaging, which results in robust findings of the type that are frequently absent in small-scale neuroimaging investigations. In fact, this may be the largest neuroimaging investigation yet performed of food reward systems in the brain and their relation to body weight/BMI. For example, activation studies to food presentation have typically involved <30 participants (Tang *et al.*, 2012; Masterson *et al.*, 2019), and previous functional connectivity studies related to eating or obesity have typically involved <100 participants (Tregellas *et al.*, 2011; Kullmann *et al.*, 2012; Lips *et al.*, 2014; Garcia-Garcia *et al.*, 2015; Donofry *et al.*, 2020; Legget *et al.*, 2021). Further, the results were cross-validated with HCP data. Moreover, the relation between the liking for food and high BMI was confirmed in an even larger investigation, involving 502 492 participants. The findings here are of special interest, for they establish an association between the resting-state functional connectivity of the extended VMPFC/OFC and the liking of the individual person for sweet foods and their BMI. A hypothesis is that the increased functional connectivity we describe here even when no food is present may be an individual difference that does influence how rewarding food is for an individual, and the increased body weight that may be related to higher eating of such foods. This hypothesis relates to the much broader hypothesis that a driving factor in evolution may be variation in the reward value of different specific types of reward in different individuals, which provides a fundamental basis of personality, that is, individual differences (Rolls, 2014). In the present case, the implication is that the variation in the connectivity of food reward systems in the brain may lead some individuals to like food more, which of course can be adaptive in some environments, and that this can in some environments, especially when food is highly palatable and readily available, be associated with a high body weight/BMI (Rolls, 2014, 2016b, 2021b).

## Supplementary Material

nsab083_Supp

## Data and code availability statement

Data for the UK Biobank are available at https://www.ukbiobank.ac.uk/ and for the Human Connectome Project at https://www.humanconnectome.org/. Standard code functions available in Matlab and SPM were used.
