# Supplementary material for: Orbitofrontal cortex connectivity is associated with food reward and body weight in humans
Source: Soc Cogn Affect Neurosci. 2021 Jul 14;18(1):nsab083. doi: 10.1093/scan/nsab083 (PMC10498940; doi:10.1093/scan/nsab083)
Supplement: nsab083_Supp [file nsab083_supp.zip › suppl.docx]

**Orbitofrontal cortex connectivity is associated with food reward and body weight in humans**

**Supplementary Material**

Social, Cognitive and Affective Neuroscience (2021)

Edmund T Rolls^1,2,3^^*^, Ruiqing Feng^1^, Wei Cheng^2^, Jianfeng Feng^1,2,4^

1. Department of Computer Science, University of Warwick, Coventry CV4 7AL, UK

2. Institute of Science and Technology for Brain-inspired Intelligence, Fudan University, Shanghai, 200433, China

3. Oxford Centre for Computational Neuroscience, Oxford, UK

4. Key Laboratory of Computational Neuroscience and Brain-Inspired Intelligence, Fudan University, Ministry of Education, Shanghai, 200433, China

**Table S1.** The anatomical regions defined in each hemisphere and their label in the automated anatomical labelling atlas AAL2 (Rolls et al., 2015). Column 4 provides a set of possible abbreviations for the anatomical descriptions.

| NO. | ANATOMICAL DESCRIPTION | LABEL  aal2.nii.gz | POSSIBLE  ABBREVIATION |
| --- | --- | --- | --- |
| 1,2 | Precentral gyrus | Precentral | PreCG |
| 3, 4 | Superior frontal gyrus, dorsolateral | Frontal_Sup | SFG |
| 5, 6 | Middle frontal gyrus | Frontal_Mid | MFG |
| 7, 8 | Inferior frontal gyrus, opercular part | Frontal_Inf_Oper | IFGoperc |
| 9, 10 | Inferior frontal gyrus, triangular part | Frontal_Inf_Tri | IFGtriang |
| 11, 12 | IFG pars orbitalis, | Frontal_Inf_Orb | IFGorb |
| 13, 14 | Rolandic operculum | Rolandic_Oper | ROL |
| 15, 16 | Supplementary motor area | Supp_Motor_Area | SMA |
| 17, 18 | Olfactory cortex | Olfactory | OLF |
| 19, 20 | Superior frontal gyrus, medial | Frontal_Sup_Med | SFGmedial |
| 21, 22 | Superior frontal gyrus, medial orbital | Frontal_Med_Orb | PFCventmed |
| 23, 24 | Gyrus rectus | Rectus | REC |
| 25, 26 | Medial orbital gyrus | OFCmed | OFCmed |
| 27, 28 | Anterior orbital gyrus | OFCant | OFCant |
| 29, 30 | Posterior orbital gyrus | OFCpost | OFCpost |
| 31, 32 | Lateral orbital gyrus | OFClat | OFClat |
| 33, 34 | Insula | Insula | INS |
| 35, 36 | Anterior cingulate & paracingulate gyri | Cingulate_Ant | ACC |
| 37, 38 | Middle cingulate & paracingulate gyri | Cingulate_Mid | MCC |
| 39, 40 | Posterior cingulate gyrus | Cingulate_Post | PCC |
| 41, 42 | Hippocampus | Hippocampus | HIP |
| 43, 44 | Parahippocampal gyrus | ParaHippocampal | PHG |
| 45, 46 | Amygdala | Amygdala | AMYG |
| 47, 48 | Calcarine fissure and surrounding cortex | Calcarine | CAL |
| 49, 50 | Cuneus | Cuneus | CUN |
| 51, 52 | Lingual gyrus | Lingual | LING |
| 53, 54 | Superior occipital gyrus | Occipital_Sup | SOG |
| 55, 56 | Middle occipital gyrus | Occipital_Mid | MOG |
| 57, 58 | Inferior occipital gyrus | Occipital_Inf | IOG |
| 59, 60 | Fusiform gyrus | Fusiform | FFG |
| 61, 62 | Postcentral gyrus | Postcentral | PoCG |
| 63, 64 | Superior parietal gyrus | Parietal_Sup | SPG |
| 65, 66 | Inferior parietal gyrus, excluding supramarginal and angular gyri | Parietal_Inf | IPG |
| 67, 68 | SupraMarginal gyrus | SupraMarginal | SMG |
| 69, 70 | Angular gyrus | Angular | ANG |
| 71, 72 | Precuneus | Precuneus | PCUN |
| 73, 74 | Paracentral lobule | Paracentral_Lobule | PCL |
| 75, 76 | Caudate nucleus | Caudate | CAU |
| 77, 78 | Lenticular nucleus, Putamen | Putamen | PUT |
| 79, 80 | Lenticular nucleus, Pallidum | Pallidum | PAL |
| 81, 82 | Thalamus | Thalamus | THA |
| 83, 84 | Heschl’s gyrus | Heschl | HES |
| 85, 86 | Superior temporal gyrus | Temporal_Sup | STG |
| 87, 88 | Temporal pole: superior temporal gyrus | Temporal_Pole_Sup | TPOsup |
| 89, 90 | Middle temporal gyrus | Temporal_Mid | MTG |
| 91, 92 | Temporal pole: middle temporal gyrus | Temporal_Pole_Mid | TPOmid |
| 93, 94 | Inferior temporal gyrus | Temporal_Inf | ITG |

Rolls, E.T., Joliot, M., Tzourio-Mazoyer, N., 2015. Implementation of a new parcellation of the orbitofrontal cortex in the automated anatomical labeling atlas. Neuroimage 122, 1-5.
